# Prognostic significance of cervical radiologic carotid artery invasion by lymph node on magnetic resonance imaging in nasopharyngeal carcinoma

**DOI:** 10.1186/s40644-023-00544-z

**Published:** 2023-03-13

**Authors:** Wenze Qiu, Xi Zhong, Jiali Jiang, Laiji Huang, Jiansheng Li, Ronghui Zheng, Zhuochen Cai, Yawei Yuan

**Affiliations:** 1 Department of Radiation Oncology, Affiliated Cancer Hospital & Institute of Guangzhou Medical University, Guangzhou, 510095 Guangdong China; 2 Department of Radiology, Affiliated Cancer Hospital & Institute of Guangzhou Medical University, Guangzhou, 510095 Guangdong China; 3 Health Ward, Affiliated Cancer Hospital & Institute of Guangzhou Medical University, Guangzhou, 510095 Guangdong China; 4 State Key Laboratory of Oncology in South China, Collaborative Innovation Center for Cancer Medicine, Guangdong Key Laboratory of Nasopharyngeal Carcinoma Diagnosis and Therapy, Sun Yat-Sen University Cancer Center, Guangzhou, 510060 Guangdong China; 5 Department of Nasopharyngeal Carcinoma, Sun Yat-Sen University Cancer Center, Guangzhou, 510060 Guangdong China

**Keywords:** Nasopharyngeal carcinoma, Carotid artery invasion, Nodal staging system, Prognostication, Magnetic resonance imaging

## Abstract

**Purpose:**

Carotid artery invasion (CAI) has been demonstrated to be an important prognosticator in some head and neck cancers. This study aimed to examine the prognostic value of radiologic CAI (rCAI) by cervical lymphadenopathy in nasopharyngeal carcinoma (NPC).

**Methods:**

NPC patients treated between January 2013 and December 2016 were included. Pre-treatment MRIs were reviewed for cervical rCAI according to the radiologic criteria. Univariate and multivariate models were constructed to assess the association between cervical rCAI and clinical outcomes. A new N classification system was proposed and compared to the 8th AJCC system.

**Results:**

The percentage of patients with MRI-positive lymph nodes was 84.7% (494/583), of whom cervical rCAI cases accounted for 42.3% (209/494). Cervical rCAI was associated with significantly poorer OS, DFS, DFFS and RFFS compared to non-rCAI (*P* < 0.05). Multivariate analyses confirmed that cervical rCAI was an independent prognosticator for DFS and DFFS, surpassing other nodal features, such as laterality, size, cervical node necrosis (CNN) and radiologic extranodal extension (rENE), while location of positive LNs remained independently associated with OS, DFS and DFFS. We propose a refined N classification: New_N1: upper neck LNs only without cervical rCAI; New_N2: upper neck LNs only with cervical rCAI; New_N3: upper and lower LNs. The proposed classification broadened the differences in OS, DFS and DFFS between N1 and N2 disease, and achieved a higher c-index for DFS and DFFS.

**Conclusions:**

Cervical rCAI was an independent unfavorable indicator of NPC. Compared to the AJCC system, the proposed N category showed satisfactory stratification between N1 and N2 disease, and better prediction of distant metastasis and disease failure.

**Supplementary Information:**

The online version contains supplementary material available at 10.1186/s40644-023-00544-z.

## Introduction

Nasopharyngeal carcinoma (NPC), a tumor arising from the epithelial cells that cover the surface and line the nasopharynx [1], is characterized by an aggressive behavior with early spread to regional lymph nodes and a high likelihood of distant metastasis [2]. Accurate assessment of cervical lymph node status is critical for guiding treatment choices and predicting prognosis in patients with head and neck squamous cell carcinoma (HNSCC), including NPC [3].

The American Joint Committee on Cancer (AJCC) staging system (8th edition) [4] for NPC classically considers a combination of lymph node (LN) factors, including laterality, size, and level. Moreover, emerging evidence has shown that other nodal features, such as cervical node necrosis (CNN) [5–7], radiologic extranodal extension (rENE) [8, 9] and more than nine MRI-positive LNs [10], may contribute to the lower survival rates in patients with metastatic nodes from cancer of the nasopharynx.

Involvement of carotid artery by a primary neoplasm or adjacent lymph nodes is not only a poor prognostic indicator but also a potential contraindication to surgical resection in HNSCC [11, 12]. As for NPC, the extension of primary tumor to carotid sheath, a part of the parapharyngeal space (PPS), is defined as T2 disease and its prognostic influence on survival outcome has been widely demonstrated [13–15]. Furthermore, Chan et al. discovered that stage II recurrent NPC encasing the internal carotid artery had significantly higher opportunity of developing subsequent systemic metastasis, even after adequate resection of the tumor [16]. However, no studies have yet examined the prognostic value of cervical carotid artery invasion (CAI) by lymph node in treatment-naive patients with NPC.

The presence of cervical CAI is chiefly assessed by imaging instead of neck dissection because, unlike other head and neck cancers, NPC is typically treated with radiotherapy. Magnetic resonance imaging (MRI) has been recommended as the optimal method for assessing T and N classification in NPC patients before treatment according to the National Cancer Comprehensive Network (NCCN) guideline. The radiologic criteria of carotid artery invasion on MRI have been previously described [11, 12, 17, 18].

Therefore, we conducted this retrospective study to comprehensively evaluate the role of radiologic CAI (rCAI) by cervical lymphadenopathy in NPC patients on the basis of MRI findings.

## Methods

### Patient population

This retrospective study evaluated 583 consecutive patients with newly diagnosed, biopsy-proven, non-metastatic NPC, who received intensity-modulated radiotherapy (IMRT) at the Affiliated Cancer Hospital & Institute of Guangzhou Medical University between January 2013 and December 2016. All patients were scanned using MRI and restaged in accordance to the 8th edition of the AJCC staging system for NPC [4]. This study was approved by the Institutional Review Boards at the Affiliated Cancer Hospital & Institute of Guangzhou Medical University. Signed informed consent was obtained from all patients before examination.

### MRI protocol

Pretreatment MRI was performed on a 1.5-T scanner with head and neck array coils. The examined area extended from the upper border of the orbit to the inferior margin of the sternal end of the clavicle. The detailed MR scanning protocol and acquisition parameters are shown in Table S1, which were described previously [19].

### Image assessment

Pre-treatment MRIs were reviewed by a radiologists (XZ) experienced in head and neck MRI blinded to clinical outcome. The diagnostic criteria for metastatic lymphadenopathy were: (1) retropharyngeal lymph nodes, cervical lymph nodes in the jugulodigastric region and any other cervical lymph nodes with a shortest axial diameter of 5 mm, 11 mm and 10 mm or greater, respectively; (2) groups of three or more borderline lymph nodes with a shortest axial diameter of 8 mm or greater; (3) lymph nodes of any size with rENE or necrosis [20, 21]. The features of rENE included the presence of indistinct nodal margins, irregular nodal capsular enhancement or infiltration into adjacent structures, such as muscles, neurovascular structures, parotid, or skin [22]. The equivocal/uncertain cases were classified as rENE-. Definition of central necrosis on MR images was a focal area of high signal intensity on T2-weighted images or a focal area of low signal intensity on T1-weighted images with or without a surrounding rim of enhancement [23].

**Fig. 1:**
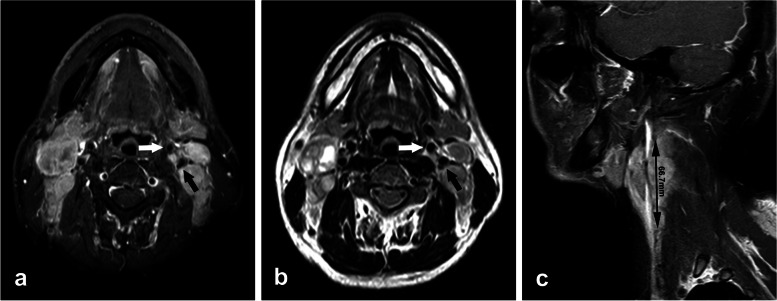
MRI scans of one patient showing lymph node metastases involving the carotid artery. **a**-**b** Axial contrast-enhanced T1WI and T2WI showing > 180 degree of encasement of the internal carotid artery with a blurred fat plane (black arrow) and < 180 degree of encasement of the external carotid artery (white arrow) at the left side. **c** Sagittal contrast-enhanced T1WI showing the length of carotid involvement at the left side, measuring 66.7 mm

Until now, no consensus has been reached on standardization of imaging criteria for defining encasement of the carotid artery. In our study, any invasion of internal, external and common carotid artery by cervical lymph node was assessed using the following radiological criteria selected from literature [11, 12, 17, 18]: (1) circumference of lymph node attachment to the artery > 180 degrees, (2) obliteration of the fat plane between the lymph node and the carotid artery, (3) deformation of the carotid artery, (4) length of contact between the carotid artery and lymph node > 30 mm. The combination of 2 or more findings was predictive of suspicious carotid artery invasion (Fig. 1).

To assess the intra/inter-rater reliability of cervical rCAI, MR scans of a subset of randomly selected cases were re-reviewed by the same radiologist (XZ) after a three-month interval and were also independently reviewed by a second radiologist (JL).

### Treatment and follow-up

The nasopharyngeal and neck tumors of all patients were treated using radical radiotherapy based on IMRT for the entire course. The primary NPC gross tumor volume was defined as the primary tumor observed during the clinical examination and imaging, and was given a total dose of 66-72 Gy in 30–33 fractions. The metastatic lymph node area was defined as the area with clinically and/or radiologically observed enlarged lymph nodes, and was given a total dose of 64-70 Gy in 30–33 fractions. During the study, institutional guidelines (based on the 7th edition AJCC cancer staging system [24]) recommended IMRT alone for stage I disease and concurrent chemoradiotherapy with or without neoadjuvant/adjuvant chemotherapy for stage II to IVB NPC. Salvage treatments (such as reirradiation, chemotherapy and surgery) were given to patients with documented relapse or persistent disease. After completion of treatment, follow-up intervals were 3 months in the first 2 years, 3–6 months for the next 3–5 years and then annually.

### Statistical analysis

Baseline characteristics were compared between the rCAI vs. non-rCAI cohorts using Chi-square, Fisher's exact, or Wilcoxon-Mann Whitney test for categorical variables. Overall survival (OS; time from the day of treatment initiation until death from any cause), disease-free survival (DFS; until failure or death from any cause, whichever occurred first), distant failure-free survival (DFFS; until distant metastasis), and regional failure-free survival (RFFS; until regional persistence/recurrence) were calculated with the Kaplan–Meier method, and differences were compared with the log-rank test. Univariate analysis was also conducted by using the log-rank test. Multivariate analysis and the effect of N stage on risk for death, disease failure and distant failure were performed by using the Cox proportional hazards model. Hazard ratios (HR) and corresponding 95% confidence intervals (CI) were calculated. Intra- and inter-observer agreements for assessing rCAI vs. non-rCAI were calculated using Cohen’s kappa test and kappa coefficient was calculated. The performances of the AJCC and the proposed N staging system were compared with the Harrell’s concordance index (c-index). Two-tailed *P* values < 0.05 were considered statistically significant. Statistical analyses were performed using the Statistical Package for the Social Sciences (SPSS) version 25.0 (IBM, Armonk, NY, USA) and R package (Version 4.0.3).

## Results

### Patient population

A total of 583 consecutive non-metastatic NPC patients were identified, of which 494 (84.7%) were lymph node-positive. The clinical characteristics of the 583 eligible patients are listed in Table 1. The male (*n* = 424)-to-female (*n* = 159) ratio was 2.7:1, and the median age was 47 years (range, 16–76 years).

**Table 1:** Clinical characteristics of 583 nasopharyngeal carcinoma patients

Variables	N0 (n = 89, 15.3%)	N + patients (*n* = 494, 84.7%)		
		Non-rCAI (*n* = 285, 57.7%)	rCAI (*n* = 209, 42.3%)	*P*
**Gender**				0.007
Male	68 (76.4)	192 (67.4)	164 (78.5)	
Female	21 (23.6)	93 (32.6)	45 (21.5)	
**Age**				0.911
≤ 47 years	45 (50.6)	146 (51.2)	106 (50.7)	
> 47 years	44 (49.4)	139 (48.8)	103 (49.3)	
**Histologic type**				0.646
KSCC	0	1 (0.4)	2 (1.0)	
NKDC	1 (1.1)	13 (4.6)	11 (5.3)	
NKUC	88 (98.9)	271 (95.0)	196 (93.7)	
**T stage**				0.701
T1	7 (7.9)	20 (7.0)	16 (7.7)	
T2	27 (30.3)	70 (24.5)	56 (26.7)	
T3	38 (42.7)	131 (46.0)	99 (47.4)	
T4	17 (19.1)	64 (22.5)	38 (18.2)	
**N stage**				< 0.001
N0	89 (100)			
N1		156 (54.7)	44 (21.1)	
N2		107 (37.5)	76 (36.4)	
N3		22 (7.8)	89 (42.5)	
**Total stage**				< 0.001
I	7 (7.9)			
II	27 (30.3)	42 (14.7)	8 (3.8)	
III	38 (42.7)	162 (56.8)	87 (41.6)	
IV	17 (19.1)	81 (28.5)	114 (54.5)	
**Chemotherapy**				0.001
RT alone	10 (11.2)			
CCRT alone	32 (36.0)	44 (15.4)	12 (5.7)	
CCRT + NAC/AC	47 (52.8)	241 (84.6)	197 (94.3)	
**Laterality of cervical positive LNs**				< 0.001
RLNs only	-	59 (20.7)	0 (0)	
Unilateral	-	100 (35.1)	74 (35.4)	
Bilateral	-	126 (44.2)	135 (64.6)	
**Location of positive LNs**				< 0.001
Upper neck only	-	265 (93.0)	143 (68.4)	
Upper + lower neck	-	20 (7.0)	66 (31.6)	
**Size of positive LNs**				< 0.001
≤ 6 cm	-	279 (97.9)	156 (74.6)	
> 6 cm	-	6 (2.1)	53 (25.4)	
**CNN**				< 0.001
No	-	236 (82.8)	87 (41.6)	
Yes	-	49 (17.2)	122 (58.4)	
**rENE**				< 0.001
No	-	157 (55.1)	4 (1.9)	
Yes	-	128 (44.9)	205 (98.1)	

Abbreviations: *rCAI* radiological carotid artery invasion, *KSCC* keratinizing squamous cell carcinoma, *NKDC* non-keratinizing differentiated carcinoma, *NKUC* non-keratinizing undifferentiated carcinoma, *RT* radiotherapy, *CCRT* concurrent chemoradiotherapy, *NAC* neoadjuvant chemotherapy, *AC* adjuvant chemotherapy, *LN* lymph node, *RLN* retropharyngeal lymph node, *CNN* cervical node necrosis, *rENE* radiological extranodal extension

The incidence of cervical rCAI in patients with positive lymph node metastases was 42.3% (209/494). The frequency of cervical rCAI increased with higher N-category: N1: 22.0% (44/200), N2: 41.5% (76/183), N3: 80.2% (89/111) and higher total stage: II: 16.0% (8/50), III: 34.9% (87/249), IV: 58.5% (114/195). Accordingly, more patients in the rCAI cohort received CCRT + NAC/AC (*P* = 0.001). Compared with the non-rCAI cohort, the rCAI cases had more bilateral and lower cervical LNs, and rCAI was found in a higher proportion of > 6 cm based on the largest LN dimension (all *P* < 0.001). In addition, LNs with rCAI were more likely to have a necrotic appearance and extranodal extension (both *P* < 0.001). Among patients with positive cervical lymph node metastases (*n* = 435), rCAI was identified at level II (37.1%, 126/340), III (44.9%, 88/196), IV (23.8%, 10/42) on the right side and level II (34.6%, 120/347), III (48.0%, 98/204), IV (23.9%, 11/46) on the left side. rCAI was not found at level I, VA or VB (Table S2).

Median follow-up for the entire cohort was 52 (range, 5–101) months. Regional failure, distant failure, disease failure and deaths were detected in 8, 96, 125 and 67, respectively. Five-year RFFS, DFFS, DFS and OS were 98.4%, 82.0%, 76.8%, and 86.2%, respectively.

### Prognostic values of cervical rCAI in 494 patients with metastatic nodes

**Fig. 2:**
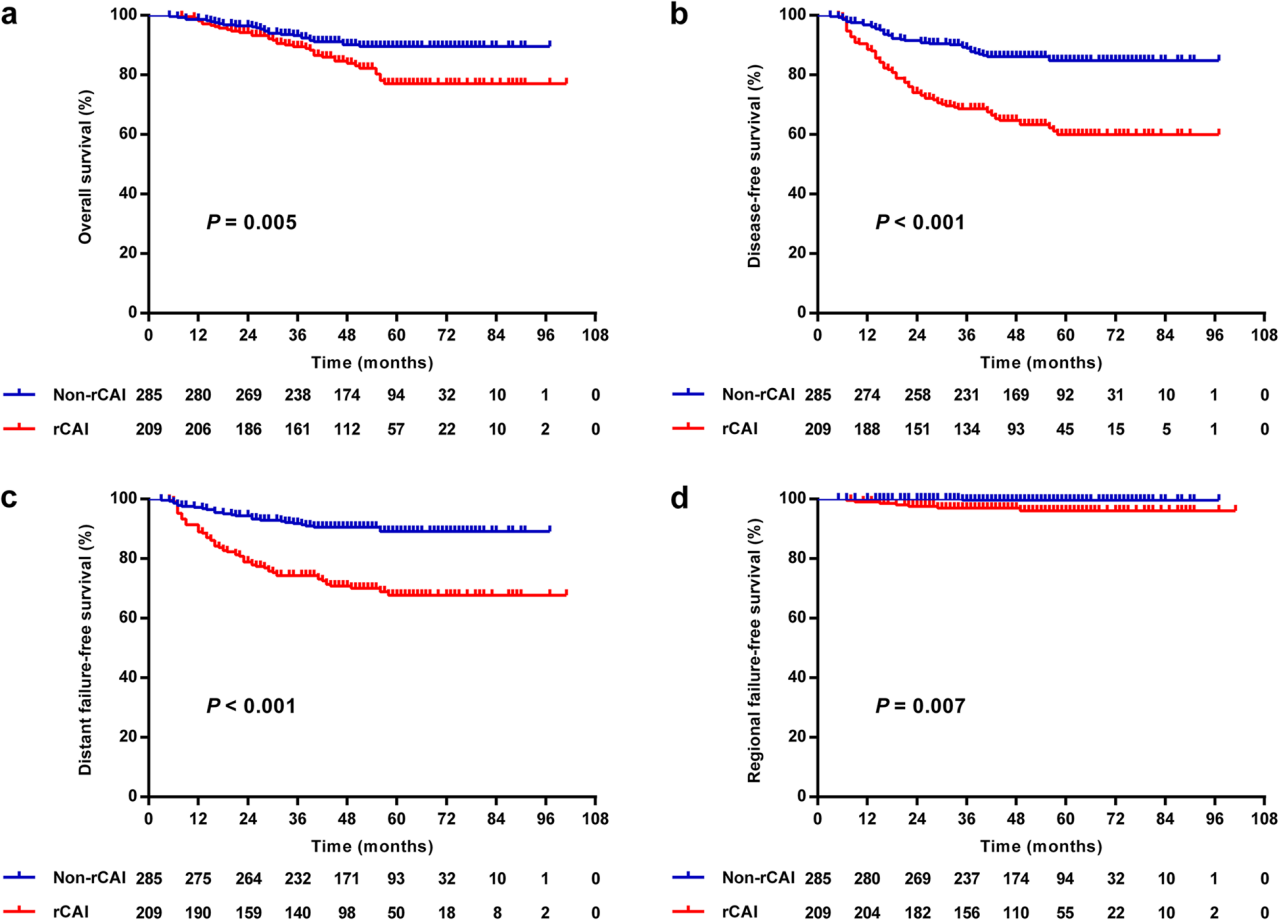
The Kaplan–Meier curves of overall **a**, disease-free **b**, distant failure-free **c** and regional failure-free **d** survival in NPC patients with or without cervical rCAI

When compared to patients with non-rCAI, the rCAI cohort had a significantly inferior 5-years OS, DFS, DFFS and RFFS (all *P* < 0.05; Table S3 and Fig. 2). In univariate analysis (Table S3), N stage, the location of positive LNs, rENE and cervical rCAI were associated with OS, DFS, DFFS and RFFS (all *P* < 0.05). Total stage, the laterality of positive LNs were related with OS, DFS and DFFS (all *P* < 0.05), but not RFFS. The size of positive LNs and CNN were in relation to DFS and DFFS (all *P* < 0.05). T stage was only associated with OS (all *P* < 0.05).

In multivariate analysis (Table 2), after adjusting for several potentially clinical confounding factors (i.e. gender, age, T stage and chemotherapy) and other nodal features (i.e. the laterality, location, size of positive LNs, CNN and rENE), cervical rCAI was independently associated with DFS (*P* = 0.010) and DFFS (*P* = 0.045), but not OS (*P* = 0.313). Additionally, location of positive LNs was an independent prognostic factor for OS (*P* = 0.007), DFS (*P* = 0.008) and DFFS (*P* = 0.012). rENE was an independent prognostic factor for OS (*P* = 0.043) and DFFS (*P* = 0.045), but not DFS (*P* = 0.053). The laterality, size of positive LNs and CNN were not independent prognostic factors for OS, DFS or DFFS.

**Table 2:** Multivariate analysis in 494 patients with metastatic nodes

Variables	OS		DFS		DFFS	
	HR (95% CI)	*P*	HR (95% CI)	*P*	HR (95% CI)	*P*
**Gender**		0.158		0.662		0.708
Male	1 (reference)		1 (reference)		1 (reference)	
Female	1.47 (0.86–2.52)		1.10 (0.72–1.66)		0.91 (0.55–1.50)	
**Age**		0.089		0.018		0.049
≤ 47 years	1 (reference)		1 (reference)		1 (reference)	
> 47 years	1.56 (0.93–2.60)		1.56 (1.08–2.26)		1.53 (1.00–2.33)	
**T stage**		< 0.001		0.011		0.067
T1	1 (reference)		1 (reference)		1 (reference)	
T2	4.42 (0.57–34.38)	0.156	2.67 (0.93–7.68)	0.069	2.10 (0.71–6.18)	0.178
T3	4.61 (0.62–34.46)	0.137	2.74 (0.98–7.66)	0.055	2.20 (0.78–6.23)	0.139
T4	19.72 (2.64–147.48)	0.004	4.71 (1.63–13.61)	0.004	3.60 (1.22–10.63)	0.021
**Chemotherapy**		0.051		0.799		0.924
CCRT alone	1 (reference)		1 (reference)		1 (reference)	
CCRT + NAC/AC	0.47 (0.22–1.00)		0.92 (0.47–1.79)		0.96 (0.43–2.14)	
**Laterality of cervical positive LNs**		0.050		0.185		0.050
RLNs only	1 (reference)		1 (reference)		1 (reference)	
Unilateral	0.47 (0.13–1.72)	0.252	0.74 (0.26–2.06)	0.559	0.41 (0.12–1.40)	0.155
Bilateral	1.02 (0.29–3.55)	0.974	1.08 (0.39–2.98)	0.882	0.72 (0.22–2.41)	0.594
**Location of positive LNs**		0.007		0.008		0.012
Upper neck only	1 (reference)		1 (reference)		1 (reference)	
Upper + lower neck	2.21 (1.24–3.93)		1.77 (1.16–2.70)		1.84 (1.14–2.96)	
**Size of positive LNs**		0.937		0.202		0.140
≤ 6 cm	1 (reference)		1 (reference)		1 (reference)	
> 6 cm	0.97 (0.44–2.12)		1.39 (0.84–2.30)		1.50 (0.88–2.58)	
**CNN**		0.468		0.586		0.637
No	1 (reference)		1 (reference)		1 (reference)	
Yes	0.81 (0.45–1.44)		0.89 (0.59–1.35)		1.12 (0.70–1.79)	
**rENE**		0.043		0.053		0.045
No	1 (reference)		1 (reference)		1 (reference)	
Yes	2.50 (1.03–6.08)		2.02 (0.99–4.12)		2.58 (1.02–6.51)	
**Cervical rCAI**		0.313		0.010		0.045
No	1 (reference)		1 (reference)		1 (reference)	
Yes	1.40 (0.73–2.70)		1.91 (1.17–3.12)		1.79 (1.01–3.17)	

Abbreviations: *OS* overall survival, *DFS* disease-free survival, *DFFS* distant failure-free survival, *HR* hazard ratio, *CI* confidence interval, *CCRT* concurrent chemoradiotherapy, *NAC* neoadjuvant chemotherapy, *AC* adjuvant chemotherapy, *LN* lymph node, *RLN* retropharyngeal lymph node, *CNN* cervical node necrosis, *rENE* radiological extranodal extension, *rCAI* radiological carotid artery invasion

### A proposed N classification system

**Fig. 3:**
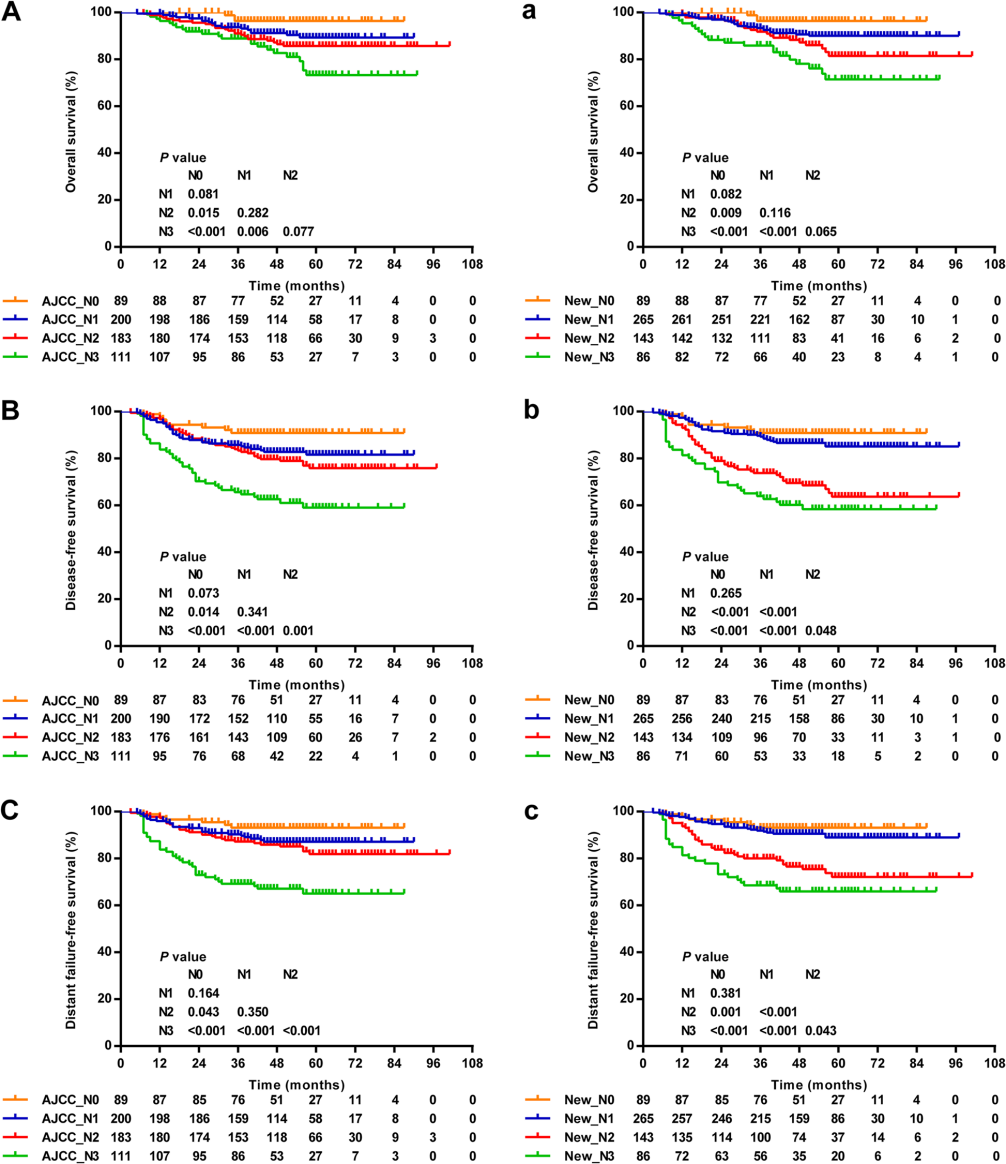
Overall (**A**, **a**), disease-free (**B**, **b**) and distant failure-free (**C**, **c**) survival for each N category as defined by the 8th edition of the AJCC N staging system (**A**-**C**) and the proposed N staging system (**a**-**c**)

Based on the results above, we proposed a revised N classification system that included the location of positive LNs and cervical rCAI: New_N1: upper neck LNs only without cervical rCAI (*n* = 265); New_N2: upper neck LNs only with cervical rCAI (*n* = 143); New_N3: upper and lower LNs (*n* = 86). A change in N3 stage mainly occurred in the 4.3% of patients (25 of 583) with > 6 cm LNs on the upper neck only, of whom 0.3% (2 of 583) without cervical rCAI were downstaged to N1 and 3.9% (23 of 583) with cervical rCAI were downstaged to N2. As shown in Fig. 3, our revised N staging system showed satisfactory stratification between classification N1 and N2 in DFS and DFFS; meanwhile, survival curves of DFS and DFFS between N2 and N3 patients remained separated significantly. However, neither of these two N category systems could significantly separate N0 and N1 in OS, DFS and DFFS. To assess the hazard ratios of the AJCC system and proposed N staging system, multivariate analysis was performed adding confounding factors of age, gender, T stage and chemotherapy. Results showed that the hazard ratio discrimination of OS, DFS and DFFS between N1 and N2 disease were improved (Table 3). Compared with the AJCC system, the c-index of the proposed N staging system showed a significant improvement for predicting DFS (0.721 vs. 0.671, *P* = 0.007), and DFFS (0.737 vs. 0.698, *P* = 0.043), but not OS (0.690 vs. 0.665, *P* = 0.177).

**Table 3:** Effect of different N stages on the risk of survival outcomes

N stage	AJCC system						Proposed system					
	OS		DFS		DFFS		OS		DFS		DFFS	
	HR (95% CI)	*P*	HR (95% CI)	*P*	HR (95% CI)	*P*	HR (95% CI)	*P*	HR (95% CI)	*P*	HR (95% CI)	*P*
N0	1 (reference)		1 (reference)		1 (reference)		1 (reference)		1 (reference)		1 (reference)	
N1	2.50 (0.72–8.71)	0.150	1.66 (0.75–3.65)	0.208	1.52 (0.61–3.79)	0.370	2.62 (0.77–8.91)	0.123	1.38 (0.63–3.03)	0.419	1.30 (0.53–3.23)	0.569
N2	4.02 (1.18–13.69)	0.026	2.29 (1.05–4.98)	0.038	2.16 (0.88–5.33)	0.093	4.39 (1.26–15.34)	0.020	3.53 (1.62–7.71)	0.002	3.39 (1.38–8.31)	0.008
N3	7.72 (2.26–26.44)	0.001	4.97 (2.28–10.83)	< 0.001	5.41 (2.22–13.16)	< 0.001	9.48 (2.75–32.69)	< 0.001	5.58 (2.52–12.38)	< 0.001	5.88 (2.37–14.61)	< 0.001

Abbreviations: *AJCC* American Joint Committee on Cancer, *OS* overall survival, *DFS* disease-free survival, *DFFS* distant failure-free survival, *HR* hazard ratio, *CI* confidence interval

### Intra- and inter-observer variation of cervical rCAI assessment

Based on a power calculation (≥ 80% power to detect significant discordance in cervical rCAI), 80 cases were randomly selected for intra- and inter-observer assessment. The intra- and inter-observer kappa for cervical rCAI was 0.812 and 0.757, respectively.

## Discussion

For head and neck squamous cell carcinoma, such as the cancer of oral cavity, oropharynx, hypopharynx and larynx, carotid artery involvement by primary tumor masses has been classified as T4b disease and is considered an unresectable disease based on the NCCN guideline [25]. For NPC, a unique type of head and neck cancer typically treated with radiotherapy, the impact of carotid artery involvement by cervical lymphadenopathy on the prognosis warrants thorough investigation.

For the first time, we observed a high incidence of cervical rCAI (42.3%) in patients with NPC and nodal metastasis. Cervical rCAI was only identified at level II, III and IV by metastatic lymph nodes, probably due to their location around the carotid artery [12, 26]. We further described the relationship between the cervical rCAI and NPC patients’ survival, and found that it is a predominant independent indicator for DFS and DFFS in NPC patients, outweighing other nodal features, such as laterality, size, CNN and rENE.

Distant metastasis has become the greatest challenge for NPC treatment owing to the substantial improvement in locoregional control [27]. A common pattern for carcinomas is that regional LNs are the first sites to develop metastases through the lymphatic pathway orderly [28]. However, the potential role of primary tumor mass or metastatic LNs as a route for hematogenous spread should not be ignored. Plenty of studies regarding the parapharyngeal space (PPS) extension have discover that distant metastasis is more likely in the presence of obvious involvement of the poststyloid compartment, which is probably due to tumor directly invading the blood vessels in the carotid sheath and increasing the risk of hematogeneous dissemination [13–15]. Similarly, in our study, metastatic LNs with CAI might denote the biological aggressiveness of cancer clones via blood stream, which facilitates systemic metastasis in NPC.

In the present study, the prognostic significance of several MRI identified nodal features, including laterality, location, size, CNN and rENE, were evaluated comprehensively. In consistent with the results of prior researches [5–9], all of the nodal factors mentioned above were associated with survival according to univariate analysis. After adjustment for potentially clinical confounding factors (i.e. gender, age, T stage and chemotherapy) and other nodal features (i.e. the laterality, location, size, CNN and rENE), multivariate analysis demonstrated that the presence of cervical rCAI still had increased HRs for distant metastasis and disease failure. Lower cervical LNs remained significant for OS, DFS and DFFS. rENE was still an independent prognostic factor for OS and DFFS. The laterality, size and CNN were not independent prognostic factors for survival any more.

This can be explained by the following reasons. ENE has been shown to contribute to disease progression in head and neck cancers, including NPC, for its tendency to increase the risk of tumor cell entering into blood stream [8, 9, 29–31]. Thus, invasion of the carotid artery, as one of the most important episodes of ENE, may reflect the impact of ENE on distant metastasis directly. Previous studies also have demonstrated that LN size and laterality seem less important in predicting prognosis when taking into consideration other nodal features, such as nodal level, or the number of positive LNs [10, 32]. The reported proportion of NPC patients with LNs greater than 6 cm based on cross-sectional imaging only ranges from 1.4% to 2.7% [10, 32]. While in our study, nodal size was measured by the largest dimension, irrespective of the measurement plane according to AJCC staging system [4], thus a higher incidence of > 6 cm LNs (11.9%; 59 of 494) was observed, which is similar to Zhou’s study (8.8%; 31 of 354) [33]. It is reasonable to speculate that larger LNs are prone to encase the artery and allow a longer contact between the lymph node mass and carotid artery, which are indirect signs of carotid invasion. Li et al. [32] found that laterality remained an independent factor for DFFS and DFS when multivariate analysis was performed in NPC patients without level IV, Vb, or supraclavicular fossa (SCF) involvement, suggesting that nodal level was of primary prognostic significance, with laterality of secondary importance.

In this study, the major limitation of the AJCC N staging system was that it could not separate survival curves between classification N1 and N2 NPC patients, which is consistent with the result of a large-scale multicenter study [34]. Intriguingly, when N2 and N1 are classified according to upper neck LNs only with or without cervical rCAI, the survival curves showed satisfactory stratification between them, with the differences of DFS and DFFS reaching statistical significance. Therefore, we propose a new N classification based on the location of positive LNs and cervical rCAI to refine AJCC_N as follows: New_N1: upper neck LNs only without cervical rCAI; New_N2: upper neck LNs only with cervical rCAI; New_N3: upper and lower LNs. On one hand, the lower level of LNs remained incorporated in the proposed staging system (New_N3) for its strong prognostic significance and proximity to the thoracic duct, which possibly mediates systemic dissemination via lymph-venous conjunction [35]. On the other hand, LNs greater than 6 cm was excluded from the N classification criteria due to its lack of significance as an independent prognostic factor. Thus, 25 patients with > 6 cm LNs on the upper neck only were downstaged to New_N1 (2 without cervical rCAI) or New_N2 (23 with cervical rCAI). The predictive power of the proposed system appeared to be improved over that of the AJCC (8th edition) staging system regarding the risk of distant metastasis and disease failure with a higher c-index. Finally, we were capable of achieving good intra-observer and inter-observer reliability for cervical rCAI, supporting consideration to include this variable in clinical N classification.

Several limitations should be noted in this study. First, all patients enrolled were from the endemic region where the predominant histology was non-keratinizing differentiated carcinoma. Whether or not cervical rCAI has the similar clinical implication in regions where keratinising carcinoma dominates is yet to be elucidated. Second, the pathologic confirmation of rCAI was unavailable in patients with NPC, who were typically treated with radiotherapy rather than surgery. Third, well-known prognostic indicators such as LN multiplicity, nodal volume and Epstein-Barr virus (EBV) DNA were not included in this study; it is yet unknown if incorporating these factors will alter the conclusions of this study. Finally, this is a retrospective study and validation of our results is needed in future data, especially in large sample, multicenter and prospective studies.

## Conclusions

In conclusion, cervical rCAI was an independent unfavorable indicator of NPC. Compared to the AJCC system, the proposed N category showed satisfactory stratification between N1 and N2 disease, and better prediction of distant metastasis and disease failure.

## Supplementary Information

**Additional file 1.** 

**Additional file 2.** 

**Additional file 3.**

## Data Availability

The datasets generated during and/or analysed during the current study are available from the corresponding author on reasonable request.

## Abbreviations

AC: Adjuvant chemotherapy
AJCC: American Joint Committee on Cancer
rCAI: Radiologic carotid artery invasion
CCRT: Concurrent chemoradiotherapy
CI: Confidence interval
c-index: Concordance index
CNN: Cervical node necrosis
DFFS: Distant failure-free survival
DFS: Disease-free survival
EBV: Epstein-Barr virus
rENE: Radiologic extranodal extension
HNSCC: Head and neck squamous cell carcinoma
HR: Hazard ratio
IMRT: Intensity-modulated radiotherapy
KSCC: Keratinizing squamous cell carcinoma
LN: Lymph node
MRI: Magnetic resonance imaging
NAC: Neoadjuvant chemotherapy
NCCN: National Cancer Comprehensive Network
NKDC: Non-keratinizing differentiated carcinoma
NKUC: Non-keratinizing undifferentiated carcinoma
NPC: Nasopharyngeal carcinoma
OS: Overall survival
PPS: Parapharyngeal space
RFFS: Regional failure-free survival
RLN: Retropharyngeal lymph node
SCF: Supraclavicular fossa
SPSS: Statistical Package for the Social Sciences
WHO: World Health Organization
