# Supplementary material for: Prognostic significance of cervical radiologic carotid artery invasion by lymph node on magnetic resonance imaging in nasopharyngeal carcinoma
Source: Cancer Imaging. 2023 Mar 13;23:26. doi: 10.1186/s40644-023-00544-z (PMC10009921; doi:10.1186/s40644-023-00544-z)
Supplement: Supplementary file 1 — Additional file 1. [file 40644_2023_544_MOESM1_ESM.docx]

**Details of MRI acquisition**

MR images were acquired using a 1.5-T system unit (Intera Achieva; Philips Healthcare, Best, The Netherlands) with a 16-channel head-neck combined coil. The MRI protocol included an axial turbo spin echo (TSE) T1-weighted sequence, an axial TSE T2-weighted sequence, a coronal short time inversion recovery (STIR) sequence, and an axial and a sagittal contrast-enhanced TSE T1-weighted sequence. Contrast-enhanced T1WI was performed after intravenous administration of 0.1 mmol/kg gadopentetate dimeglumine (Magnevist, Bayer Schering). Details of the MRI acquisition were showed in **Table S1**.

**Table S1** MRI sequences and parameters

| **Sequence** | **FS** | **TR/TE (ms)** | **Gap (mm)** | **ST (mm)** | **FOV (cm)** | **Matrix** |
| --- | --- | --- | --- | --- | --- | --- |
| Axial TSE T1-weighted | No | 545/14 | 4 | 4 | 23 | 328 × 220 |
| Axial TSE T2-weighted | No | 3193/80 | 5 | 5 | 23 | 228 × 185 |
| Coronal STIR | Yes | 3224/165 | 5 | 5 | 26 | 312 × 163 |
| Axial contrast-enhanced TSE T1-weighted | Yes | 545/14 | 4 | 4 | 23 | 328 × 220 |
| Sagittal contrast-enhanced TSE T1-weighted | Yes | 545/14 | 4 | 4 | 26 | 328 × 220 |

Abbreviations: FS, fat suppression; TR, repetition time; TE, echo time; STIR, short time inversion recovery ; ST, slice thickness; FOV, field of view.
