# Supplementary material for: Prognostic significance of cervical radiologic carotid artery invasion by lymph node on magnetic resonance imaging in nasopharyngeal carcinoma
Source: Cancer Imaging. 2023 Mar 13;23:26. doi: 10.1186/s40644-023-00544-z (PMC10009921; doi:10.1186/s40644-023-00544-z)
Supplement: Supplementary file 2 — Additional file 2. [file 40644_2023_544_MOESM2_ESM.docx]

**Table S2 The incidence of rCAI at different** **cervical nodal levels**

| Nodal level | Cervical LN+ patients (n=435) | | | | | | |
| --- | --- | --- | --- | --- | --- | --- | --- |
|  | Right side | | |  | Left side | | |
|  | LN+ | rCAI+ | rCAI+/LN+ (%) |  | LN+ | rCAI+ | rCAI+/LN+ (%) |
| Level I | 22 | 0 | 0 |  | 18 | 0 | 0 |
| Level II | 340 | 126 | 37.1 |  | 347 | 120 | 34.6 |
| Level III | 196 | 88 | 44.9 |  | 204 | 98 | 48.0 |
| Level IV | 42 | 10 | 23.8 |  | 46 | 11 | 23.9 |
| Level VA | 89 | 0 | 0 |  | 106 | 0 | 0 |
| Level VB | 18 | 0 | 0 |  | 12 | 0 | 0 |

Abbreviations: rCAI, radiologic carotid artery invasion; LN: lymph node.
