# Supplementary material for: Prognostic significance of cervical radiologic carotid artery invasion by lymph node on magnetic resonance imaging in nasopharyngeal carcinoma
Source: Cancer Imaging. 2023 Mar 13;23:26. doi: 10.1186/s40644-023-00544-z (PMC10009921; doi:10.1186/s40644-023-00544-z)
Supplement: Supplementary file 3 — Additional file 3. [file 40644_2023_544_MOESM3_ESM.docx]

**Table S3 Univariate analysis in 494 patients with metastatic nodes**

| Variables | 5-Year survival rate (%) | | | | | | | |
| --- | --- | --- | --- | --- | --- | --- | --- | --- |
|  | OS | *P* | DFS | *P* | DFFS | *P* | RFFS | *P* |
| **Gender** |  | 0.362 |  | 0.792 |  | 0.287 |  | 0.855 |
| Male | 85.7 |  | 73.7 |  | 78.6 |  | 98.0 |  |
| Female | 81.1 |  | 75.9 |  | 83.8 |  | 98.5 |  |
| **Age** |  | 0.611 |  | 0.064 |  | 0.118 |  | 0.884 |
| ≤47 years | 84.6 |  | 77.9 |  | 82.7 |  | 98.4 |  |
| >47 years | 84.4 |  | 70.5 |  | 77.3 |  | 97.8 |  |
| **Histologic type** |  | 0.320 |  | 0.142 |  | 0.509 |  | 0.604 |
| KSCC | 66.7 |  | 66.7 |  | 66.7 |  | 100 |  |
| NKDC | 82.5 |  | 74.3 |  | 86.3 |  | 95.8 |  |
| NKUC | 84.6 |  | 74.5 |  | 79.8 |  | 98.2 |  |
| **T stage** |  | <0.001 |  | 0.085 |  | 0.259 |  | 0.834 |
| T1 | 97.2 |  | 88.8 |  | 88.8 |  | 97.2 |  |
| T2 | 88.0 |  | 74.6 |  | 80.5 |  | 99.0 |  |
| T3 | 88.8 |  | 74.9 |  | 80.5 |  | 97.9 |  |
| T4 | 65.4 |  | 67.1 |  | 75.2 |  | 97.8 |  |
| **N stage** |  | 0.022 |  | <0.001 |  | <0.001 |  | 0.016 |
| N1 | 89.3 |  | 81.6 |  | 87.2 |  | 99.5 |  |
| N2 | 85.7 |  | 75.9 |  | 81.9 |  | 98.9 |  |
| N3 | 73.3 |  | 59.0 |  | 65.0 |  | 94.1 |  |
| **Total stage** |  | <0.001 |  | <0.001 |  | <0.001 |  | 0.292 |
| II | 92.0 |  | 89.8 |  | 93.8 |  | 100 |  |
| III | 91.7 |  | 79.8 |  | 85.3 |  | 98.8 |  |
| IV | 73.0 |  | 63.3 |  | 69.8 |  | 96.6 |  |
| **Chemotherapy** |  | 0.671 |  | 0.207 |  | 0.187 |  | 0.302 |
| CCRT alone | 81.3 |  | 81.6 |  | 86.7 |  | 100 |  |
| CCRT+NAC/AC | 85.0 |  | 73.3 |  | 79.2 |  | 97.9 |  |
| **Laterality of cervical positive LNs** |  | 0.004 |  | 0.004 |  | 0.003 |  | 0.369 |
| RLNs only | 92.5 |  | 88.9 |  | 90.6 |  | 100 |  |
| Unilateral | 91.3 |  | 78.5 |  | 85.4 |  | 98.8 |  |
| Bilateral | 78.4 |  | 68.4 |  | 74.2 |  | 97.2 |  |
| **Location of positive LNs** |  | 0.001 |  | <0.001 |  | <0.001 |  | <0.001 |
| Upper neck only | 87.1 |  | 77.7 |  | 83.1 |  | 99.2 |  |
| Upper+lower neck | 71.5 |  | 58.4 |  | 65.9 |  | 92.3 |  |
| **Size of positive LNs** |  | 0.742 |  | 0.002 |  | <0.001 |  | 0.915 |
| ≤6 cm | 84.8 |  | 76.5 |  | 82.6 |  | 98.1 |  |
| >6 cm | 80.9 |  | 56.9 |  | 59.8 |  | 98.2 |  |
| **CNN** |  | 0.262 |  | 0.008 |  | 0.001 |  | 0.837 |
| No | 86.1 |  | 78.4 |  | 84.8 |  | 98.1 |  |
| Yes | 81.2 |  | 66.6 |  | 71.2 |  | 98.2 |  |
| **rENE** |  | 0.007 |  | <0.001 |  | <0.001 |  | 0.041 |
| No | 91.3 |  | 88.3 |  | 92.2 |  | 100 |  |
| Yes | 81.0 |  | 67.6 |  | 74.2 |  | 97.1 |  |
| **Cervical rCAI** |  | 0.005 |  | <0.001 |  | <0.001 |  | 0.007 |
| No | 89.5 |  | 84.8 |  | 89.1 |  | 99.6 |  |
| Yes | 77.0 |  | 59.9 |  | 67.7 |  | 96.0 |  |

Abbreviations: OS, overall survival; DFS, disease-free survival; DFFS, distant failure-free survival; RFFS, regional failure-free survival; KSCC, keratinizing squamous cell carcinoma; NKDC, non-keratinizing differentiated carcinoma; NKUC, non-keratinizing undifferentiated carcinoma; CCRT, concurrent chemoradiotherapy; NAC, neoadjuvant chemotherapy; AC, adjuvant chemotherapy; LN: lymph node; RLN: retropharyngeal lymph node; CNN, cervical node necrosis; rENE, radiological extranodal extension; rCAI, radiological carotid artery invasion.
